# A Practical Treatise on Porrigo, or Scald Head, and on Impetigo, the Humid, or Running Tetter, with Plates

**Published:** 1816-04

**Authors:** 


### PART II.

### COMPREHENSIVE ANALYTICAL REVIEW

OF

### MEDICAL LITERATURE.

"Tros, tyriusve, *nobis* nullo discrimine agetur."

*A Practical Treatise on Porrigo, or Scald Head, and on Impetigo, the humid, or running Tetter, with Plates.* By the late ROBERT WILLAN, M. D. Edited by ASHBY SMITH, M. R. C. S. pp. 48. 4to.

The Manuscript of this Work was committed to Mr. Smith's care, shortly before Dr. Willan's decease at Madeira, in 1812. It is published as the author left it, with the exception of a few notes. The subjects though limited in number, are in themselves complete; and of considerable importance. The greater part of the work is occupied with the description and treatment of PORRIGO; a daily increasing, and loathsome malady, which is too justly reckoned among the *Opprobria Medicorum.*

Porrigo is characterised as a contagious disease without fever; usually exhibiting an eruption of pustules, termed Achores and Favi. The Achor, is a small accuminated pustule, which contains a straw coloured fluid, having the appearance, and nearly the consistence of strained honey; appearing most frequently on the head, and succeeded by a dull white, or yellowish scab. The FAVUS is larger, and less elevated than the Achor; being succeeded by a yellow, semitransparent, and sometimes cellular scab, like a honey-comb.

The varieties of Porrigo are — P. larvalis — P. furfurosa — P. lupinosa — P. scutulata — P. decalvans — P. favosa. The P. larvalis (crusta lactea) generally appears on the forehead, in minute pustules, with a whitish point, set close together, and producing a redness and inequality of the surface, attended with considerable itching. The pustules break in a few days, and discharge a clear, viscid humor, which gradually concretes into thin yellowish scabs. From beneath these, a fluid is discharged from time to time, forming additional layers of scab, of a brown, or blackish colour, till the forehead is completely incrusted. The scabs are sometimes thick and rounded, though not compact; at others, thin and laminated with loose edges. They do not separate at regular periods; when detached, the surface is soon covered with a new incrustation. The scab is alternately dry and humid. The discharge is sometimes so profuse as to leave the surface in a state of ulceration, emitting a thin acrimonious fluid from innumerable pores. This, in young infants, causes extreme pain, itching, and irritation. When the disease is about to terminate, the scab becomes dry; falls off, and leaves a red, shining cuticle, indented with deep lines, and very brittle. Hence it cracks and exfoliates: being sometimes renewed three or four times before the surface becomes natural. This complaint sometimes appears first on the hairy scalp; at others, on the cheeks, chin, temples, or ears; but wherever it commences, the eruption usually extends, in the course of a few months, to all the parts abovementioned, and likewise to the neck or breast, so that the whole face looks as if covered with a vizor; the nose and eyelids alone being exempt from the dark incrustations. The fluid that distils from among the scabs, diffuses a rank, unpleasant smell, and is very acrimonious excoriating the contiguous parts. Dr. W. has seen it affect various parts of the body.

An eruption of numerous small Achores is succeeded by layers of brown or blackish laminated scabs, which after a few weeks, become dry, and whitish, and at length, falling off, discover a red, smooth, and shining cuticle. A repetitition of this series often takes place. The symptoms are milder in proportion to the advanced age of the child. In infants, a few months after birth, a long series of other disorders is frequently superadded to Porrigo. The eyes become inflamed, as also the cilia, with purulent discharge. The ears frequently discharge; the parotid and submaxillary glands become hard, and suppurate. The same thing happens occasionally to the glands of the groin and axilla. In some instances of protraction, the abdomen becomes hard and tumid, and hectic fever with diarrhœa concludes the scene. Others seem to die of the exhaustion produced by the profuse discharge from among the scabs and glands, or from the pain and irritation consequent thereon. Adult men are seldom, if ever, affected with this complaint; but in boys and women, it sometimes appears on the forehead, temples, and cheeks,

The duration of P. larvalis in infants, is often considerable; from three to eighteen months, during which, the situation and appearance vary much. The incrustation is sometimes removed by the measles, but returns. It is sometimes so inveterate as to resist every application for five or six months; but it is consoling, that, whatever be the *temporary*, it is not followed by permanent deformity. Dr. W. thinks, that lactation has no influence on the complaint.

"While the discharge continues acrid and profuse, all the parts affected should be gently washed or sponged, at least twice a day, with some emollient liquid: I generally direct a decoction of bran, strained and warm. Ointments cannot be properly applied over the whole surface of the body: the unguentum zinci may, however, be put on places that are tender, and liable to excoriation, as the prominent part of the forehead, and of the cheeks, the ears, the chin, and the hollows of the joints. Moderate doses of the submurias hydrargyri should be administered twice a day, for three or four weeks at a time. It acts better in some cases if it be made into a powder with carbonate of soda, and a few grains of testaceous powder, more especially when there is a defect in the biliary secretion, with induration of the mesenteric glands, as stated page 7. In delicate infants, whose bowels are too irritable to bear these medicines, the washed calomel (hydrarg. oxyd. ciner.) or the hydrargyrus cum creta may be advantageously substituted. Where mercurials are not strongly indicated, the sulphur præcip. combined with alkaline or testaceous powder, lessens the discharge, and contributes to remove the irritation on the skin. As soon as the scabs appear dry, and begin to separate, the ointment prescribed below,* may be applied to the edges of them twice a day; and if no internal malady intervene, the powder or decoction of Peruvian bark will be found beneficial. When the face and scalp are covered with a moist scab, and where the eyes or eyelids are inflamed, blisters are generally recommended. I have applied them, in many cases, between the shoulders, with less success than has been promised, or might reasonably have been expected.

Professor Strack recommends a decoction of the viola tricolor, or pansie, as an infallible remedy in this disease. A handful of the fresh leaves boiled for some minutes in half a pint of cow's milk, to be taken morning and evening.

2. *Porrigo furfurosa,* Chiefly affects adults; first appearing uniformly diffused over the greater part of the

---

* Ung. ceruss. acet. ung. ceræ. Pharm prioris — Ung. hyd. nit. aa partes æqual. misce pro ung.

scalp, sometimes encroaching on the forehead, temples, and cheeks. The scabs soon become superficially dry, and powdery, easily separable, and quickly re-produced. Great quantities come away with the comb, attended with itching and tenderness of the scalp: often with exquisite pain; when the tops of the pustules, or portions of the incrustations are detached. Women are most subject to P. furfurosa; which weakens, thins, and sometimes alters the colour of the hair; circumstances distinguishing it from Pityriasis.

Before any applications are made to the scalp in this disorder, the head should be shaved, and the scab gently removed by warm water and soap. The following ointments are then useful, viz. Ung. zinci. — ung. hydrarg. nitrat. — ung. hyd. nit. oxyd. — ung. picis. — ung. sulph. &c. Dr. W. prefers an ointment with the *cocculus indicus* finely powdered: first suggested to him by Dr. James Hamilton, of Edinb. The proportion is one part of the powdered nut to three parts of lard. After two or three applications of this ointment, the head will admit of being shaved. The ointment should be applied night and morning, and the parts frequently washed with soap and water. The use of an oil skin cap is necessary during the cure.

3. Porrigo Lupinosa. Small separate clusters of Achores are first formed on the different parts, which presently break, and form dry, yellowish, or white scabs, which in a fortnight increase to the size of a sixpence, with a raised border, and a depressed furrowed centre. Intervening white incrustations nearly cover the scalp, falling off in small white laminæ. If neglected, the accumulating scab forms with the hair into a hard, elevated mass, resembling a bird's nest, or honey-comb. It is usually of long duration; not confined to the scalp, but appearing on the arms, neck, and thighs, in small distinct cavities, with a raised edge, and uneven surface, containing a white scaly powder. After the removal of these cup-like scabs by suitable applications, the cuticle beneath appears dry, red, shining, and porous, the surface not becoming smooth in less than five or six weeks, When the scalp is affected, it is necessary to remove the hair and employ frequent ablutions. Where the hardened incrustations are impenetrable, either by ointment, or by soap and hot water, a lotion made with diluted muriatic acid, will generally prove effectual in clearing the surface, and the unguentum *cocculi indici* may be afterwards applied with success. If the skin be dry and hard, this ointment does not act so well as the unguentum picis.

Dr. W. has prescribed the sulphuret of antimony, antimonial and mercurial preparations, sulphur, sarsa, &c. with little advantage.

"Cleanliness, good diet, and proper exercise, seem, in this complaint to be the best means of producing a favourable change on the constitution." p. 17.

4. Porrigo Scutulata is characterised by extensive circular blotches on the scalp, neck, &c. at first consisting of distinct straw-coloured pustules, which presently break, and form separate rounded scabs. After their removal, the surface appears red, shining, and slightly papulated. In a week or two, a fresh eruption of pustules comes out on the same places. The circular areas then increase, and the succeeding scabs are more thick and diffuse. The blotches continue to enlarge to considerable dimensions. During their extension, the hairs within the circles appear of a lighter colour, and sometimes break off about an inch from the scalp, and the bulbs are occasionally destroyed. If neglected, the circular blotches coalesce, and the hair is finally extirpated. The scalp then reassumes its natural appearance. A border of hair is usually left uninjured round the head.

*The Porrigo Scutulata* affects children of two years and upwards; continuing in many cases for a series of years; and is the most unmanageable species of all. Its victims are often pale, languid, emaciated, and subject to induration of the glands. It is highly contagious; and often communicated by hair dressers using the same combs, with different people.

"While the blotches and the adjoining scalp in this complaint are red, or inflamed and very tender, the whole surface should be sponged twice a day with warm water, or some mild fomentation, and afterwards covered by light clean linen caps. All irritating applications, at first, tend to aggravate; the weekly shaving, though absolutely necessary for the preservation of the hair, cannot be performed without pain and difficulty. By degrees the inflammation subsides, and desquamation takes place, as well as scabbing. Afterwards, in the repeated eruptions which take place, the blotches, in some cases, become again red and inflamed; in others, they are irritative with little inflammation, but with an acrid discharge; and in others, languid and inert, with a scaly scab, the appearance of them being only changed by the application of very powerful stimulants. Under these varying circumstances, how futile must be the pretensions of those who profess to remove this complaint, and every other form of Porrigo by one specific application. The following remedies, or some modification of them, may be employed according to the difference of appearances. Ointments prepared with the *cocculi indici*, tobacco, or opium; the ceratum lythargyri acet. — ung zinci. — ung hyd. nit. oxid. — ung. hyd. præcip. alb. — ung. hyd. nit. — ung. hyd. submur. — ung. sulph. — picis. — elemi — hellebori — oxym. hyd. — sulph cup, &c. In acknowledging the utility of this list of remedies, I must observe, that practitioners will seldom succeed by trusting the cure of the Porrigo scutulata to any of them singly; nor can he be sure, whatever modifications he may have successfully adopted in one case, that the process will answer in another." Blisters sometimes give a temporary relief. The violent eradication of the hair does the same.

5. Porrigo Decalvans. Here the hairs suddenly fall off, leaving bald patches, which are neither inflamed nor discoloured. The smooth shining appearance of the scalp, without redness or ulceration, sufficiently distinguishes this complaint from the preceding. It sometimes affects adults. "All the varieties of Porrigo may be produced from the same contagion," one form predominating among assemblages of children, the other forms appearing in individuals.

When Porrigo decalvans is neglected, the patches remain bald for several weeks, but hair at length re-appears on them, though differing from the rest in colour and texture. In adults it will be entirely grey; in children of a light brown hue.

"These alterations may, in general, be entirely prevented by repeatedly shaving the whole scalp, and by applying warm liniments. I have used naphtha, and oil of tar, also slight tinctures of the oil of mace, of camphor, and tobacco." 23

6. Porrigo Favosa, is an eruption of the pustules termed Favi, on the head, face, and other parts of the body. On the *scalp*, the pustules are large, soft, whitish, itching, and slightly inflamed at the base. At first distinct, and partially distributed, as on the side of the head, or about the occiput. When broken, they discharge a thick, viscid matter, which gradually concretes into irregular brown, or yellowish semi-transparent scabs. The ulcerations gradually extend with a constant and copious discharge, by which the scabs are kept moist, and the hairs are matted together. Pediculi then breed plentifully; produce incessant irritation, and contribute to aggravate the disease. The eruptions finally cover the whole scalp; the pustules in some places distinct, in others, confluent, forming irregular ulcerated blotches; whence a sour, rancid vapour is exhaled. In many cases, there are among the pustules, small, red, smooth tumors, which desquamate at the top, and gradually suppurate. Sometimes large abscesses form near the vertex, and while these discharge freely, the pustular eruption disappears.

During the course of P. favosa on the scalp, the glands of the neck, and sometimes the parotids inflame, harden, and even suppurate.

Porrigo favosa on the *face*, sometimes commences about the lips, or upon the chin; but at other times extends thither from the scalp, or from behind the ears. The pustules in general appear first at the corner of the mouth, set near together in irregular clusters, containing a straw coloured fluid.

When broken, they become confluent; discharge a clear viscid matter, which concretes into a yellowish scab. Similar ulcerations soon appear on the lips, or about the chin. The blotches being attended with an incessant itching, children keep their edges raw and inflamed by continually picking them. The complaint has a most unpleasant aspect, when the ulcerations entirely surround the mouth. The matter discharged is very acrimonious, and when transmitted along the lymphatics, inflames the absorbent glands. Children from the ages of one to five years, are most liable to P. favosa on the face. In almost every case, the eruption is attended with a discharge from behind the ears, with swelling and hardness of the upper lip; with enlargement, and often ulceration of the glands about the neck or throat; and with inflammation of the eyes, or obstinate ulceration of the ciliary glands. The duration of the complaint is very uncertain. Adults are occasionally affected with this form.

The P. favosa affects the arms, back, thighs, ancles and feet, in distinct blotches, similar to those on the face, and often contemporaneous with them. Deep and obstinate ulcerations are formed at the root of the toes, on the ankles, and heels. Dr. W. has seen the complaint diffused over the whole body of infants under one year old; and particularly severe. The eruption consists of Achores partially mixed with the Favi. There is not much inflammation round the pustules; but after they are broken, a considerable discharge takes place, of a clear, unctuous fluid, gradually concreting into irregular yellow scabs. As the eruption proceeds, the inguinal, axillary, and perhaps cervical glands become hard and enlarged; some of them finally inflame and suppurate.

Infants suffer much from the pain and itching of this complaint; often for two or three months together; fresh pustules continually rising on the places whence the scabs had been removed. Dr. Willan says nothing of the treatment, but concludes with a quotation from Underwood, who says

"It has always been benefited for a while, by washing the parts with two drachms of the aqua kali puri, in a pint of water."

The internal remedies recommended in the other species of Porrigo, must here be had recourse to; as also the external applications enumerated before.

We have here given our readers a full view of Dr. Willan's account of this disease, as far as verbal description will go. Three plates are dedicated to the elucidation of Porrigo, which are neatly executed.

### Impetigo, or the humid running Tetter.

By the term Impetigo, Dr. W. means to express an eruption of pustules usually denominated Psydracia. The Psydraceum is a minute pustule, irregularly circumscribed, producing slight elevation of the cuticle, and terminating in a laminated scab. Many of these pustules usually appear together, and become confluent. When mature, they contain pus; after breaking, they discharge a thin watery humour. These pustules, when the eruption is extensive, do not appear uniform. Some are small and elevated; others larger, and less elevated: a few resemble vesicles, being filled with a semi-transparent fluid. The impetigo is neither contagious, nor communicable by inoculation. Its varieties are — 1. Impetigo Sparsa. 2. I. figurata. 3. I. Erysipelatodes. 4. I. Scabida. 5. I. Rodens.

1. In Impetigo Sparsa the pustules are at a distance from each other, and the eruption extends, without any certain order, along the backs of the hands, the arms, neck, shoulders, thighs, or legs. After a few days, the pustules break and discharge a thin humour, gradually concreting into thin laminated scabs. The cuticle becomes reddish, rough, or scaly, with slight discharges from chaps in various places. The duration of the complaint in the upper extremities is seldom more than two or three weeks. On the lower extremities it is longer. Here small yellow pustules first appear on the instep, then on the ancle and leg, with violent itching. When broken, a quantity of humour issues out from small pores, around which the cuticle is rough, reddish, and a little elevated. The parts affected are, for some weeks, covered with thin scabs; but not so as to prevent the watery discharge. When all appears to be healing, a fresh eruption of pustules, and discharge take place, with great heat and irritation. After several such eruptions, ulcers are formed about the ancle, discharging a clear ichor, and exhibiting unequal cavities and irregular edges, surrounded by pustules. In old and sedentary patients, the edges of the ulcers are black or purple; the limbs œdematous. The Impetigo sparsa is most troublesome when the yellow psydracia are intermixed with small irregular vesicles, especially on the upper extremities. The complaint commences about the knuckles, and spreads along the thumb and fingers to the nails; also along the back of the hand, and round the wrists to the fore-arm. The eruption extends, in some cases, to the bend of the elbows, the upper arm, the neck, and the cheek, succeeded by a watery discharge and the formation of laminated scabs. When these fall off, the cuticle beneath remains for a long time scaly and chapped, fresh pustules arising from time to time, with heat, soreness, and violent tingling. Thus, by repeated suppuration and scabbing, the texture of the skin becomes, in many places, rough, harsh, and inflexible.

This disease generally appears in autumn, and continues through the greater part of the succeeding winter. It disappears, in many cases, during the summer; but returns at the latter end of the season. The eruption is preceded by some disorder of the constitution, as head-ache, indigestion, and pains in the stomach; violent pains in the limbs and back, and sometimes cramps of the lower extremities. Adults are most subject to this disease. There is an hereditary predisposition to it. The complaint appears after intemperance, violent exercise, or exposure to too sudden interchanges of heat and cold.

2. Impetigo figurata exhibits blotches a little elevated, and distinctly circumscribed, but of different forms, circular, oval, angulated, or tortuous. The round and angular appear chiefly on the arm, small, and at a distance from each other, extending, in many cases, to the back of the hand. Some appear even on the cheeks, forehead, and chin. On the lower extremities, large, oval, or irregular blotches take place on the instep, leg, and outside of the thigh. Every blotch is at first formed by a cluster of psydracia, set close together, and filled with yellow matter. These breaking, a thin irritating humour is discharged from minute orifices, by the concretion of which, a yellowish scab is formed, often intersected by painful fissures, from which a little moisture continues to exude. In two or three weeks the discharge ceases, the scabs dry and fall off, and the cuticle is left red, harsh, and elevated. Excoriation is easily produced in this state of the skin, so that the ichorous discharge and scabbing recommence, and the disorder is protracted for several months.

The Impetigo figurata is generally preceded in this country by pains of the stomach, head, and a sensation of languor. It begins in the spring, and continues several months, usually returning every year about the same time, though not in the same degree.

3. The Impetigo Erysipelatodes is attended with a redness and general swelling of the face, and œdema of the eye-lids. It differs from erys. phlegmon. in producing little disorder of the constitution, in being of much longer duration, and in exhibiting an eruption of psydracia instead of large irregular bullæ. The pustules, which are numerous, appear first on the cheek, and afterwards extend over the whole face, sometimes to the neck and breast. The inflammation causes a continuous redness, tumefaction, heat, and tingling. When the pustules break, they discharge copiously an ichorous fluid; the whole surface, in ten or twelve days, being covered with thin yellowish scabs. In this manner the disease is continued for about a month, when the surface becomes dry, red, brittle, and scaly, as at the termination of other species of impetigo.

4. In the Impetigo Scabida, some of the limbs are covered with a thick, hard, continuous scab, not unlike the rough bark of trees. At the commencement of this disease, numerous psydracia are diffused over the arms and legs. The eruption, in the upper extremities, extends round the fleshy part of the fore arm, and sometimes along the back of the hand to the fingers. New pustules arise daily, but soon break, discharging a thin, acrimonious humour, till nearly the whole surface is ulcerated. At the end of a month, the discharge abates, and all the arms, from the elbow to the wrist, is invested with a thick, rugged, yellowish-brown scab. When this appearance has continued for some time, extensive fissures are usually formed, and from them a watery fluid exudes, which, by its concretion, produces additional layers of scab. The disorder is constantly attended with a disagreeable sensation of heat and itching; also with pain and stiffness in the motion of the arms. If any portion of the scab fall off, or should be forcibly detached, the surface presently ulcerates again, and the breach is filled up by the discharge concreting into new scabs. When the pustules are numerous on the hand, the fingers become incrusted and rigid, and the nails gradually separate: those which succeed are thick, uneven, or notched, and often encurvated.

The progress of Impetigo scabida on the lower extremities does not differ essentially from the above.

5. Impetigo Rodens. In this, the cellular membrane is affected as well as the skin. The pustules appear in a large irregular cluster, on the side, near the breast, and the eruption extends slowly over the breast, in some cases, to the axilla, or below the spine; in others, over the shoulders down the back. The pustules are often intermixed with vesicles, both of which break in two or three days after their appearance, and discharge an acrid humour, which continues to flow for a long time; and without destroying the skin, deeply injures the cellular texture of the part. If the breast, it gradually shrinks till nearly the whole substance of it be removed. As the scabs dry off, we find red, tender, and depressed cicatrices of temporary duration, broken up in a week or two by a fresh eruption, the affected part becoming more and more deeply corroded.

Dr. W. never saw this disease terminate but with life. Very little advantage has resulted from the attempts made to cure it by either external or internal remedies. Opium in considerable doses is the only medicine which has afforded a temporary relief from pain. The Impetigo rodens appears, in some cases, to originate from long-continued ill health; in others, from hereditary predisposition.

"TREATMENT.

"The IMPETIGO SPARSA may be removed, in a few weeks, by diligently washing the parts affected with warm water, or bran and water; and by taking sulphur, or some of the preparations of it. Returns of this eruption seem to be prevented, in many persons, by sea-bathing, and by the use of the Harrogate water. Dr. Underwood says, that such eruptions affecting children, 'yield, in a short time, to the expressed juice of the Sium aquaticum, mixed with an equal quantity of new milk.'—Vol. I. p. 95.

When the papulæ and their interstices are livid, as mentioned, p. 36-7, much advantage may be derived from Peruvian bark and muriatic acid.

"In the Impetigo figurata, I have prescribed, with success, Plummer's pill and the decoction of elm bark, or of sarsaparilla. The ung. zinci and the ceratum lyth. acetati comp are proper external applications. The following list of remedies for the impetiginous ring-worm in a hot climate, is given by Dr. Dancer:— Ung. sulphuris, sulphur bath, vervain, dumb cane, or arum seguinum, cassia occidentalis, and cassia herpetica. The bruised leaves, or expressed juice, of the vegetables mentioned, are chiefly employed: the juice of the cassia herpetica (ring-worm bush), he says, is accounted a slight escharotic. He adds, that local tetters or ring-worms, on the hands, face, and arms, are cured by the application of ink, gunpowder, and lime-juice, or ketchup. The dilating impetiginous blotches covered with a scab, as stated, page 39, may be removed by Plummer's pill, taken with the above decoction, and by applying cloths, constantly wetted with a solution of the submuriate of quicksilver in lime-water."

"The Impetigo erysipelatodes is alleviated by administering, soon after its commencement, two or three gentle purgatives, and afterwards the Peruvian bark in powder, or a strong decoction of it with sarsaparilla. In the Impetigo scabida, before the proper applications can be made to the arm or leg, it is necessary to soften and gradually remove the thick, rugged incrustation. This cannot always be accomplished by washing or fomenting, but we generally succeed by exposing the limb, for some minutes every day, to the steam of hot water. When the surface is cleared, it should be washed night and morning with warm water, and should afterwards be covered with oiled silk, a mild ointment being interposed, such as the ung. zinci, or ung. hydrarg. nitrat. lowered by the addition of three parts of unguent. ceræ. Plummer's pill, and the decoctum ulmi, dulcamaræ, &c. will also be found very advantageous in this form of impetigo, but the sulphur water, at Harrogate, &c. is greatly preferable to any internal remedy with which we are yet acquainted." 48.

We have thus exhibited a full and complete analysis of the volume before us; which, as far as verbal descriptions can go, will be found to convey an accurate idea of the diseases in question. The 1st plate exhibits an excellent view of Porrigo larvalis, and also of Porrigo favosa affecting the face. The 2d represents Porrigo scutulata as it affects the scalp. The 3d. Porrigo favosa affecting the scalp. The 4th Impetigo sparsa in an incipient stage—2d. figure, Impetigo figurata on the arm. The 5th. presents a good view of that loathsome disease Impetigo Scabida, and Impetigo Sparsa.

